# PTEN and NEDD4 in Human Breast Carcinoma

**DOI:** 10.1007/s12253-015-9971-2

**Published:** 2015-08-15

**Authors:** Yilun Chen, Marc J. van de Vijver, Hanina Hibshoosh, Ramon Parsons, Lao H. Saal

**Affiliations:** Division of Oncology and Pathology, Department of Clinical Sciences, Lund University, Lund, Sweden; Lund University Cancer Center, Lund, Sweden; Department of Pathology, Academic Medical Center, Amsterdam, The Netherlands; Department of Pathology, Columbia University Medical Center, NY, USA; Department of Oncological Sciences, Icahn School of Medicine at Mount Sinai, NY, USA; CREATE Health Strategic Centre for Translational Cancer Research, Lund University, Lund, Sweden

**Keywords:** PTEN, NEDD4, Breast carcinoma, IHC

## Abstract

PTEN is an important tumor suppressor gene that antagonizes the oncogenic PI3K/AKT signaling pathway and has functions in the nucleus for maintaining genome integrity. Although PTEN inactivation by mutation is infrequent in breast cancer, transcript and protein levels are deficient in >25 % of cases. The E3 ubiquitin ligase NEDD4 (also known as NEDD4-1) has been reported to negatively regulate PTEN protein levels through poly-ubiquitination and proteolysis in carcinomas of the prostate, lung, and bladder, but its effect on PTEN in the breast has not been studied extensively. To investigate whether NEDD4 contributes to low PTEN levels in human breast cancer, we analyzed the expression of these proteins by immunohistochemistry across a large Swedish cohort of breast tumor specimens, and their transcript expression levels by microarrays. For both NEDD4 and PTEN, their transcript expression was significantly correlated to their protein expression. However, comparing NEDD4 expression to PTEN expression, either no association or a positive correlation was observed at the protein and transcript levels. This unexpected observation was further corroborated in two independent breast cancer cohorts from The Netherlands Cancer Institute and The Cancer Genome Atlas. Our results suggest that NEDD4 is not responsible for the frequent down-regulation of the PTEN protein in human breast carcinoma.

## Introduction

PTEN is a phosphatase that plays an important role in tumor suppression by negatively regulating the oncogenic phosphatidylinositol 3-kinase (PI3K) pathway, as well as through functions in the nucleus that contribute to maintenance of genomic integrity [1]. Germline mutations of PTEN are found in patients with PTEN hamartoma tumor syndrome and are associated with an increased risk for breast, thyroid, and endometrial cancer [2–4]. Moreover, somatic loss-of-function mutations of *PTEN* are estimated to be present in 30 % of cancer and are found across the entire spectrum of tumor types [5–7]. The PTEN/PI3K pathway is one of the key pathways deregulated in breast cancer. *PIK3CA*, which encodes the p110-alpha catalytic subunit of PI3K, has activating mutations in one-third of breast tumors, and although mutation rate of *PTEN* is less than 5 % [8], PTEN expression is found to be greatly diminished in at least 25 % of breast tumors and in near mutual exclusivity to *PIK3CA* mutation [9, 10]. The mechanisms by which PTEN is down-regulated is poorly delineated in breast cancer, but mutations, copy number loss, rearrangements, epigenetic silencing, as well as post-translational regulation may contribute [9–13]. Of note, PTEN loss is frequent within the poor-prognosis basal-like molecular subtype of breast cancer [13].

Recently, Wang et al*.* reported that NEDD4 (neural precursor cell expressed, developmentally down-regulated 4, E3 ubiquitin protein ligase; also known as NEDD4-1) is an E3 ubiquitin ligase of PTEN and catalyzes poly-ubiquitination of PTEN in cells leading to proteolysis of the PTEN protein, thereby negatively regulating PTEN abundance [14]. Furthermore, in their analysis of mouse prostate and human bladder cancer samples, high expression of NEDD4 was inversely correlated to PTEN protein levels but not *PTEN* mRNA levels, suggesting that NEDD4 plays a proto-oncogenic role in tumorigenesis and cancer development via post-translational suppression of PTEN [14]. Negative regulation of PTEN by NEDD4-mediated poly-ubiquitination has since been reported to be involved in several biological and pathological processes, such as axon branching [15, 16], T-cell activation [17], keloid formation [18], and insulin-mediated glucose metabolism [19]. Inverse relationships between the expression of NEDD4 and PTEN have also been observed in human non-small cell lung carcinomas [20] and colon cancer [21].

However, the regulation of PTEN by NEDD4 may be microenvironment and/or cell-type specific. For example, Trotman et al. found that in addition to catalyzing poly-ubiquitination of PTEN, NEDD4 is also responsible for PTEN mono-ubiquitination that leads to PTEN nuclear import and protection from proteasomal degradation, making the role of NEDD4 in regulation of PTEN stability subtle and complex [22]. Moreover, some studies have called into question the interaction between NEDD4 and PTEN. For example, no discernible effect on Pten stability, subcellular localization, or downstream targets was observed in two separate Nedd4 knock-out mouse models [23]. Furthermore, Maddika et al*.* failed to reproduce the functional interaction between NEDD4 and PTEN, and instead found that WWP2, another E3 ubiquitin ligase within the NEDD4-like protein family, mediated poly-ubiquitination of PTEN [24]. A third group has also failed to demonstrate that PTEN is a substrate of Nedd4, and rather found that PTEN regulated Nedd4 by modulating mTORC1 activity [19]. Lastly, in gastric carcinoma, no relationship was observed between NEDD4 and PTEN expression [25], and in colorectal cancer cell lines and biopsies, NEDD4 modulation and expression level were not associated to the levels of PTEN [26].

NEDD4 and its potential role in PTEN regulation in breast cancer have not been studied. To reveal the pattern of expression of NEDD4 in human breast cancer, and to investigate whether NEDD4-mediated PTEN degradation is a factor that contributes to the frequent loss of PTEN protein, we analyzed NEDD4 and PTEN expression at the protein and mRNA levels in a large cohort of Swedish breast tumors, and verified our findings in two independent breast cancer cohorts from The Netherlands Cancer Institute (NKI) and The Cancer Genome Atlas (TCGA) (Table 1).

**Table 1:** Clinical demographics of the breast cancer patients

	Swedish Cohort						NKI cohort		TCGA cohort	
	With protein data				With mRNA data					
	*n* = 186 (%)		*n* = 123 (%)		*n* = 105 (%)		*n* = 295 (%)		*n* = 970 (%)	
Median age at diagnosis (y/o)	62	(range, 26–80)	64	(range, 31–80)	61	(range, 26–77)	44	(range, 26–53)	59	(range, 26–90)
Median tumor size (mm)	25	(range, 2–55)	25	(range, 10–55)	27	(range, 2–50)	20	(range, 2–50)	NA	(NA)
Estrogen receptor										
Positive	121	(65)	85	(69)	55	(52)	214	(73)	716	(74)
Negative	59	(32)	35	(28)	47	(45)	72	(24)	210	(22)
Unknown	6	(3)	3	(2)	3	(3)	9	(3)	44	(5)
Progesterone receptor										
Positive	78	(42)	55	(45)	35	(33)	185	(63)	622	(64)
Negative	98	(53)	64	(52)	62	(59)	101	(34)	301	(31)
Unknown	10	(5)	4	(3)	8	(8)	9	(3)	47	(5)
HER2										
Positive	27	(15)	16	(13)	18	(17)	56	(19)	148	(15)
Negative	113	(61)	84	(68)	55	(52)	217	(74)	496	(51)
Equivocal	NA	(NA)	NA	(NA)	NA	(NA)	NA	(NA)	156	(16)
Unknown	46	(25)	23	(19)	32	(30)	22	(7)	170	(18)
Nottingham histological grade										
1	3	(2)	1	(1)	3	(3)	60	(20)	NA	(NA)
2	47	(25)	15	(12)	37	(35)	99	(34)	NA	(NA)
3	37	(20)	14	(11)	28	(27)	136	(46)	NA	(NA)
Unknown	99	(53)	93	(75)	37	(35)	0	(0)	NA	(NA)
Lymph node										
Positive	118	(63)	79	(64)	65	(62)	144	(49)	411	(42)
Negative	68	(37)	44	(36)	40	(38)	151	(51)	397	(41)
Unknown	0	(0)	0	(0)	0	(0)	0	(0)	162	(17)

## Materials and Methods

### Breast Cancer Cohorts

Clinical and demographic information is provided for all cohorts in Table 1. For the Swedish cohort, 132 formalin-fixed paraffin-embedded (FFPE) tissue microarray (TMA) tumor specimens, arrayed in triplicates, were studied for NEDD4 protein expression by IHC, of which 123 had matched PTEN IHC scores previously evaluated [9, 27]. These 123 samples were analyzed for correlation between PTEN and NEDD4 protein levels. Correlation between the PTEN protein and *NEDD4* mRNA levels, and correlation between *PTEN* mRNA and *NEDD4* mRNA levels were analyzed in a subset of 105 samples with both PTEN IHC status and microarray gene expression data [27] (NCBI Gene Expression Omnibus accession GSE5325). Correlation between NEDD4 protein and *NEDD4* mRNA levels was performed in a subset of 42 samples with NEDD4 IHC and microarray data. For the NKI cohort, gene expression microarray data from 295 tumor samples was analyzed for correlation between gene expression levels of *PTEN* and *NEDD4* [28, 29]. Tissue microarrays containing these 295 NKI cases were stained for PTEN protein, of which 267 samples could be evaluated, and thereafter were analyzed for correlations between PTEN IHC scores and *PTEN* or *NEDD4* mRNA expression levels. For TCGA cohort, level 3 IlluminaHiSeq_RNASeqV2 gene expression data for 970 primary breast tumor samples was used, as well as PTEN protein expression status for 407 cases derived from a reverse phase protein arrays platform. All TCGA data were downloaded from the TCGA data portal (https://tcga-data.nci.nih.gov/tcga/, downloaded on January 20, 2014). The study was approved by the Lund University Hospital ethics committee (LU240-01 and 2009/658), waiving the requirement for informed consent for the study, and all experimental protocols were performed in accordance with approved guidelines.

### Immunohistochemistry

**Fig. 1:**
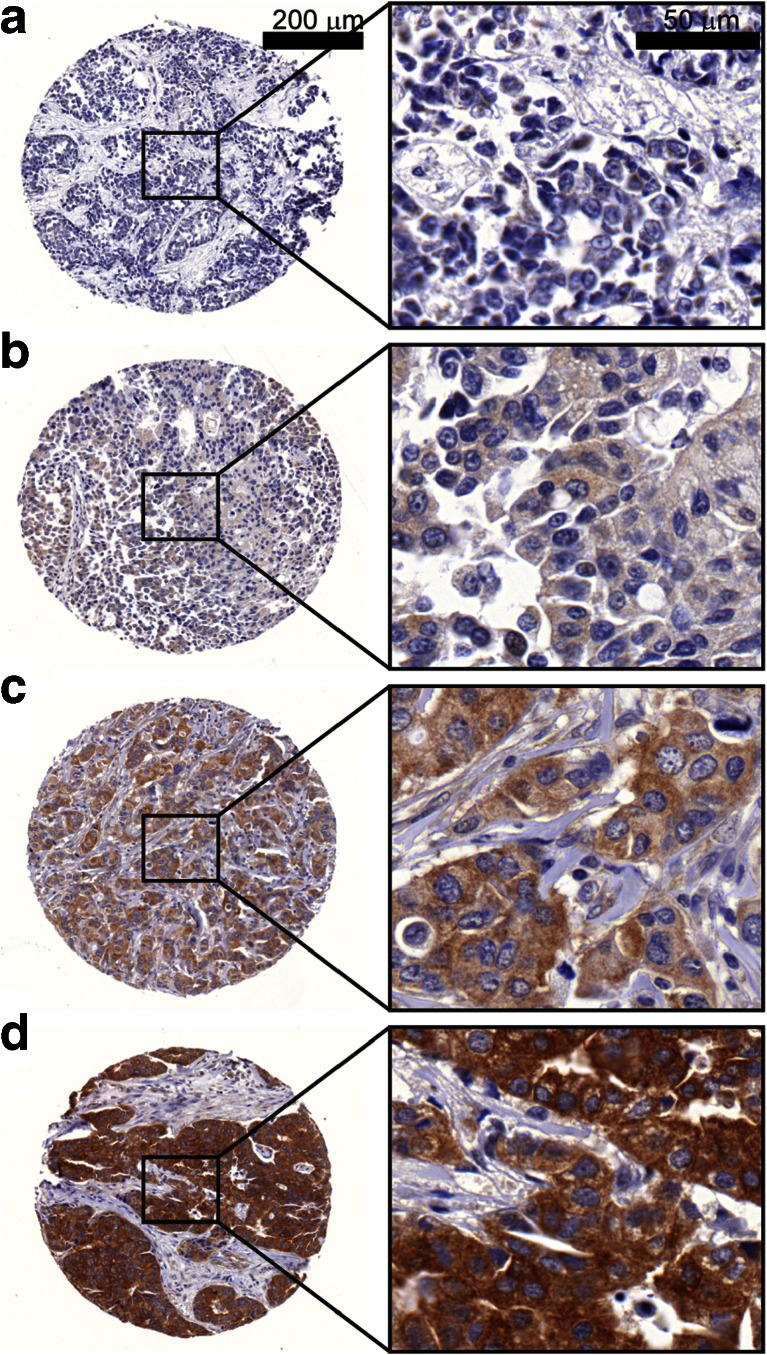
NEDD4 immunohistochemistry. 132 breast tumor tissue microarray specimens were immunohistochemically stained with anti-NEDD4 antibody. Shown are representative examples of tumors with NEDD4 IHC scores of **a** 0, **b** 1+, **c** 2+, and **d** 3+. Scores 0/1+ were categorized NEDD4-negative, and 2+/3+ as NEDD4-positive. NEDD4 protein was expressed predominantly in the cytoplasm regardless of the staining intensity

The rabbit polyclonal anti-NEDD4 WW2 domain antibody #07–049 (EMD Millipore, Darmstadt, Germany), previously validated to be specific for NEDD4 [14], was used for IHC. The staining was done using an Autostainer Plus instrument and EnVision Plus system (Dako Denmark A/S, Glostrup, Denmark) following manufacturer’s recommended protocol. Antigen retrieval was performed using Dako Targeted Retrieval Buffer pH 6.0 at 98 °C for 20 min, and the primary antibody was used at 1:500 dilution with 30 min incubation time at room temperature. The stained specimens were scanned using a MIRAX MIDI slide scanner (Carl Zeiss AG, Oberkochen, Germany) and viewed with Pannoramic Viewer v1.15.3 (3DHISTECH, Budapest, Hungary). Semi-quantitative scoring was done according to the Dako system 0–3 scoring scale, where scores of 0 were given to tissues with no NEDD4 staining, 1+ to weak NEDD4 staining, 2+ to intermediate NEDD4 staining, and 3+ to strong NEDD4 staining (Fig. 1). The IHC scores of 0 and 1+ were then combined and categorized as NEDD4-negative, and scores of 2+ and 3+ were categorized as NEDD4-positive. PTEN IHC results for the Swedish cohort were reported previously [9, 27]. PTEN IHC was performed on the NKI TMAs using methods previously described [13].

### Statistical Analysis

The chi-squared test was used to test the significance level of correlations between the NEDD4 protein and different breast cancer biomarkers. The Wilcoxon rank-sum test was used for correlation between PTEN and NEDD4 protein levels. The Student’s t-test was used for correlations between the PTEN/NEDD4 protein and *PTEN*/*NEDD4* mRNA levels. The Pearson’s correlation test was used for correlations between *PTEN* and *NEDD4* mRNA levels from gene expression data and RNA-seq data. All tests were two-tailed, and *P* < 0.05 was considered significant. All statistical analyses were performed with R version 3.1.0 (http://www.r-project.org).

## Results and Discussion

**Fig. 2:**
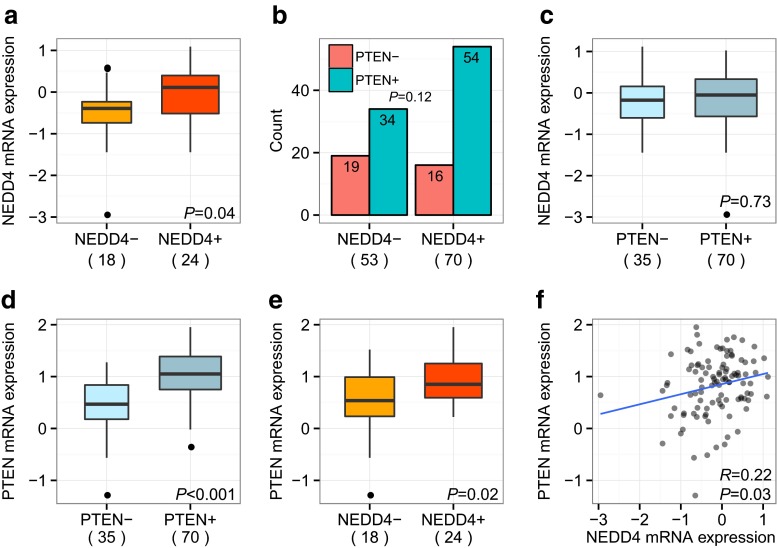
PTEN and NEDD4 protein and mRNA levels in the Swedish cohort. **a** NEDD4 protein levels were significantly correlated to *NEDD4* mRNA levels (N = 42, *P* = 0.04). PTEN protein levels were not significantly correlated to **b** NEDD4 protein levels in breast cancer tissues (N = 123, *P* = 0.12) or **c** *NEDD4* mRNA levels (N = 105, *P* = 0.73). *PTEN* mRNA levels were significantly correlated to **d** PTEN protein levels (N = 105, *P* < 0.001), **e** NEDD4 protein levels (N = 42, *P* = 0.02), and **f** *NEDD4* mRNA levels (N = 105, *R* = 0.22, *P* = 0.03)

Immunohistochemical (IHC) staining was performed for 132 formalin-fixed paraffin-embedded (FFPE) breast tumor specimens (Swedish cohort) using an antibody previously reported to be specific to NEDD4 [14] (see Methods; Fig. 1). Consistent with previous studies in other tissues [14], NEDD4 protein was predominantly cytoplasmic in breast cancer cells (Fig. 1). Among the 132 stained samples, 60 (45 %) had zero or weak NEDD4 protein staining (classified as NEDD4-negative), whereas 72 (55 %) had intermediate to strong expression (NEDD4-positive). NEDD4 protein expression was positively correlated to estrogen receptor status (ER; *P* = 0.0017), but not associated to the other clinical variables progesterone receptor (PR; *P* = 0.12), human epidermal growth factor receptor 2 (HER2; *P* = 0.12), Nottingham Histologic Grade (*P* = 0.57), and Ki-67 (*P* = 0.40) (Table 2). Microarray gene expression data were available for 42 of the 132 cases from a previous study [27]. Using this data, we found NEDD4 protein levels to be significantly correlated to *NEDD4* mRNA expression level (*P* = 0.04) (Fig. 2a), supporting the specificity of the antibody and also indicating that *NEDD4* mRNA may be an appropriate surrogate for NEDD4 protein levels in breast cancer.

**Table 2:** Correlations of NEDD4 protein with biomarkers in the Swedish cohort

	NEDD4-	NEDD4+	N	χ^2^ *P*
Estrogen receptor				
Positive	33	57	129	0.0017
Negative	26	13		
Progesterone receptor				
Positive	21	36	128	0.12
Negative	36	35		
HER2				
Positive	11	6	108	0.12
Negative	40	51		
Nottingham histological grade				
1	0	1	32	0.4
2	9	7		
3	10	5		
Ki-67				
Positive	2	7	37	0.57
Negative	9	19

PTEN protein expression was previously determined by IHC for 123 of the 132 cases [9]. We tested whether NEDD4 protein levels were negatively associated to PTEN protein levels, however no correlation was seen in this Swedish breast cancer material (*P* = 0.12; Fig. 2b). This was inconsistent with the inverse correlation between the two proteins observed in a mouse prostate cancer model [14] and in lung cancers [20]. In fact, in our Swedish cohort the correlation trended positively, with 77 % of cases being PTEN-positive within the NEDD4-positive group compared to 64 % being PTEN-positive in the NEDD4-negative group (Fig. 2b).

In human bladder carcinoma, Wang et al*.* reported *NEDD4* mRNA expression and *PTEN* mRNA expression to be uncorrelated, but that *NEDD4* mRNA levels were inversely correlated to PTEN protein levels [14]. To investigate if it is also the case in breast tumors, we next considered the transcript levels of these genes using the GSE5325 microarray dataset of 105 breast tumors previously utilized to develop a gene expression signature for PTEN-loss [27]. In contrast to bladder cancer, we found no correlation between *NEDD4* mRNA and PTEN protein expression (*P* = 0.73; Fig. 2c). *PTEN* mRNA, however, was highly correlated to PTEN protein (*P* < 0.001; Fig. 2d), which has been previously reported [27]. Unexpectedly, we found *PTEN* mRNA levels to be significantly positively correlated to NEDD4 protein expression (N = 42, *P* = 0.02; Fig. 2e) as well as to *NEDD4* mRNA levels (N = 105, *P* = 0.03; Fig. 2f).

**Fig. 3:**
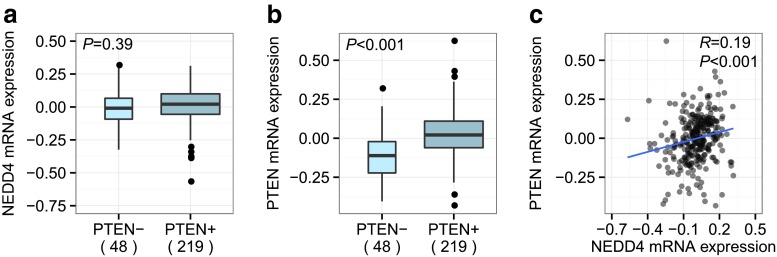
PTEN mRNA/protein levels and *NEDD4* mRNA levels in the NKI cohort. PTEN IHC scores were not associated to **a** *NEDD4* mRNA levels (N = 267, *P* = 0.39), but were significantly correlated to **b** *PTEN* mRNA levels (N = 267, *P* < 0.001). **c** *PTEN* mRNA and *NEDD4* mRNA levels were also significantly correlated (N = 295, *R* = 0.19, *P* < 0.001)

To validate these findings, two independent large-scale breast cancer cohorts from the NKI and TCGA were studied. The NKI cohort contained 295 breast tumor samples with microarray gene expression data [28, 29]. Tissue microarray sections were obtained and immunostained for PTEN protein, of which 267 cases were evaluable. Similar to the Swedish cohort, we found no correlation between *NEDD4* mRNA and PTEN protein (*P* = 0.39; Fig. 3a). The strong positive correlation between *PTEN* mRNA and PTEN protein (*P* < 0.001; Fig. 3b), as well as the association of our previously published PTEN-loss signature [27] with loss of PTEN protein (*P* = 0.003; data not shown), were confirmed in this independent dataset. Moreover, the positive association between *NEDD4* mRNA and *PTEN* mRNA found in our Swedish cohort was also validated in the NKI patient material (N = 295, *P* < 0.001; Fig. 3c).

**Fig. 4:**
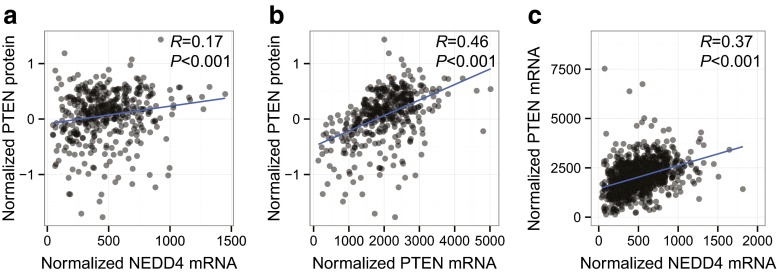
*PTEN* and *NEDD4* mRNA levels in the TCGA cohort. PTEN protein levels were significantly correlated to **a** *NEDD4* mRNA levels (N = 407, *R* = 0.17, *P* < 0.001), and **b** *PTEN* mRNA levels (N = 407, *R* = 0.46, *P* < 0.001). **c** *PTEN* mRNA levels were significantly correlated to *NEDD4* mRNA levels in the 970 primary breast tumors (*R* = 0.37, *P* < 0.001)

These associations were further corroborated in the TCGA breast carcinoma cohort containing RNA-sequencing (RNA-seq) gene expression profiles of primary breast tumors from 970 patients, of which 407 also had available PTEN protein expression data derived from reverse phase protein arrays [10]. In this large cohort the correlation between *NEDD4* mRNA and PTEN protein was also significantly positive (*P* < 0.001; Fig. 4a). Additionally, *PTEN* mRNA and PTEN protein levels were positively correlated (*P* < 0.001; Fig. 4b), as observed in the Swedish and NKI cohorts. Lastly, the positive correlation between *NEDD4* mRNA and *PTEN* mRNA levels was also confirmed in the TCGA dataset (*P* < 0.001; Fig. 4c).

In conclusion, our study investigated whether PTEN was associated to NEDD4 in three large independent breast cancer sample cohorts. Contrary to reports in some other cancer forms, no inverse relationship was seen between *NEDD4* transcript and PTEN protein levels. Rather, there was no correlation between NEDD4 protein and PTEN protein, and the correlation between NEDD4 mRNA/protein and *PTEN* mRNA was significantly positive. NEDD4-mediated poly-ubiquitination of PTEN may be an important mechanism that contributes to PTEN protein loss in bladder cancer [14] and non-small cell lung carcinoma [20]; whereas the results in gastric and colorectal cancers have been discrepant [25, 26]. Interestingly, in ovarian cancer HeLa cells, PTEN has also been reported to negatively regulate NEDD4 expression via the PI3K/AKT pathway, forming a potential negative feedback loop [30]. Our present study does not support NEDD4 as a major negative regulator of PTEN levels in human breast cancer. Additional studies are necessary to better delineate the underlying mechanisms of PTEN loss in this poor-prognosis subgroup.
